# Reversal of the Inflammatory Responses in Fabry Patient iPSC-Derived Cardiovascular Endothelial Cells by CRISPR/Cas9-Corrected Mutation

**DOI:** 10.3390/ijms22052381

**Published:** 2021-02-27

**Authors:** Hui-Yung Song, Yi-Ping Yang, Yueh Chien, Wei-Yi Lai, Yi-Ying Lin, Shih-Jie Chou, Mong-Lien Wang, Chien-Ying Wang, Hsin-Bang Leu, Wen-Chung Yu, Chian-Shiu Chien

**Affiliations:** 1 Central Region Laboratory, Center for Diagnostics and Vaccine Development, Centers for Disease Control, Ministry of Health and Welfare, Taipei 11561, Taiwan; shy770307@gmail.com; 2 Division of Basic Research, Department of Medical Research, Taipei Veterans General Hospital, Taipei 11217, Taiwan; molly0103@gmail.com (Y.-P.Y.); g39005005@gmail.com (Y.C.); jefflai8228@gmail.com (W.-Y.L.); s19609005@gm.ym.edu.tw (Y.-Y.L.); ohyeahchou@gmail.com (S.-J.C.); monglien@gmail.com (M.-L.W.); 3 Institute of Pharmacology, National Yang-Ming University, Taipei 11221, Taiwan; hsinbangleu@gmail.com; 4 Institute of Pharmacology, National Yang-Ming Chiao Tung University, Hsinchu 30010, Taiwan; 5 Department of Internal Medicine, Taipei Veterans General Hospital, Taipei 11217, Taiwan; wangcywang5363@gmail.com (C.-Y.W.); wcyu@vghtpe.gov.tw (W.-C.Y.); 6 Critical Center, Taipei Veterans General Hospital, Taipei 11217, Taiwan; 7 School of Medicine, National Yang-Ming University, Taipei 11221, Taiwan; 8 School of Medicine, National Yang-Ming Chiao Tung University, Hsinchu 30010, Taiwan; 9 Institute of Food Safety and Health Risk Assessment, National Yang-Ming University, Taipei 11221, Taiwan; 10 Institute of Food Safety and Health Risk Assessment, National Yang-Ming Chiao Tung University, Hsinchu 30010, Taiwan

**Keywords:** induced pluripotent stem cells, Fabry disease, CRISPR/Cas9 gene editing, inflammatory

## Abstract

The late-onset type of Fabry disease (FD) with *GLA* IVS4 + 919G > A mutation has been shown to lead to cardiovascular dysfunctions. In order to eliminate variations in other aspects of the genetic background, we established the isogenic control of induced pluripotent stem cells (iPSCs) for the identification of the pathogenetic factors for FD phenotypes through CRISPR/Cas9 genomic editing. We adopted droplet digital PCR (ddPCR) to efficiently capture mutational events, thus enabling isolation of the corrected FD from FD-iPSCs. Both of these exhibited the characteristics of pluripotency and phenotypic plasticity, and they can be differentiated into endothelial cells (ECs). We demonstrated the phenotypic abnormalities in FD iPSC-derived ECs (FD-ECs), including intracellular Gb3 accumulation, autophagic flux impairment, and reactive oxygen species (ROS) production, and these abnormalities were rescued in isogenic control iPSC-derived ECs (corrected FD-ECs). Microarray profiling revealed that corrected FD-derived endothelial cells reversed the enrichment of genes in the pro-inflammatory pathway and validated the downregulation of NF-κB and the MAPK signaling pathway. Our findings highlighted the critical role of ECs in FD-associated vascular dysfunctions by establishing a reliable isogenic control and providing information on potential cellular targets to reduce the morbidity and mortality of FD patients with vascular complications.

## 1. Introduction

Fabry disease (FD) is the second most frequent lysosomal storage disorder caused by a progressive accumulation of globotriaosylceramide (Gb3) in body fluids and tissues due to deficient α-galactosidase A (GLA) activity [1]. The classical phenotype of FD without any GLA activity presents a wide range of systemic symptoms, particularly manifesting as renal and cardiovascular dysfunctions [2,3]. In contrast, a milder form of FD with residual GLA activity does not exhibit classical manifestations, except for the late-onset cardiomyopathy, and this has been identified as a cardiac variant type of FD [4,5]. In this cardiac variant type of FD, a novel intronic mutation at intron 4 that contains a single G > A mutation at genomic 9331 (IVS4 + 919G→A) and an insertion of 57 nucleotides between exon 4 and 5 of the GLA transcript [6] and it has been reported with a high incidence in the cardiac variant of FD in Taiwan (about 1 in 1500 males) [7,8,9,10,11,12]. Fabry cardiomyopathy, such as left ventricular hypertrophy, is known as the predominant morbidity of FD [13]. In addition, FD patients demonstrated increased intima-media thickness (IMT) and dilation of systemic arteries with a disturbed flow pattern, suggesting an early onset of vascular disorder [14]. There is evidence indicating that the vascular lesion formation in FD occurs as a result of endothelial cell dysfunction due to abundant Gb3 accumulation, which leads to altered cerebral perfusion and a pro-thrombotic phenotype [15]. In our previous works, we have generated FD patient-derived induced pluripotent stem cells (FD-iPSCs) and differentiated them into cardiomyocytes and endothelial cells [16,17]. FD-iPSC-derived cardiomyocytes exhibited remarkable cardiomyocyte hypertrophy, lysosomal abnormalities, Gb3 deposition (IL18, Alox12/15), and aberrant electrophysiology [16]. FD-iPSC-derived endothelial cells also carry abnormal Gb3 accumulation and exhibit increased levels of reactive oxygen species and low expression of mitochondrial superoxide dismutase 2 [17]. In order to better understand the underlying pathogenesis mechanism for FD vasculopathy, we employed induced pluripotent stem cell (iPSC) technologies to establish a disease-in-a-dish model for FD-related vascular endothelial dysfunction that allows the investigation of FD’s vasculopathy that cannot be recapitulated in animal models [17,18]. RNA-guided clustered regularly interspaced short palindromic repeats (CRISPR) is a powerful genetic editing tool to knock in a template to correct the mutation in iPSCs by homologous recombination (HR) [19]. In this study, we utilized FD patient-derived iPSCs bearing the *GLA* IVS4 + 919G > A mutation combined with gene editing, endothelial differentiation, and genomics technologies to identify pathways leading to vascular dysregulation in FD. We examined the efficacy of CRISPR-mediated correction of the GLA IVS4 + 919G > A mutation in the mutated genotype and the FD-related phenotypes in iPSC-derived endothelial cells. These results demonstrated that genomic editing-based intervention modulated the inflammatory pathways and repaired the FD-specific phenotypes.

## 2. Results

### 2.1. Correction of Fabry GLA IVS4 + 919 G > A Mutation in Isogenic Control iPSCs

**Figure 1:**
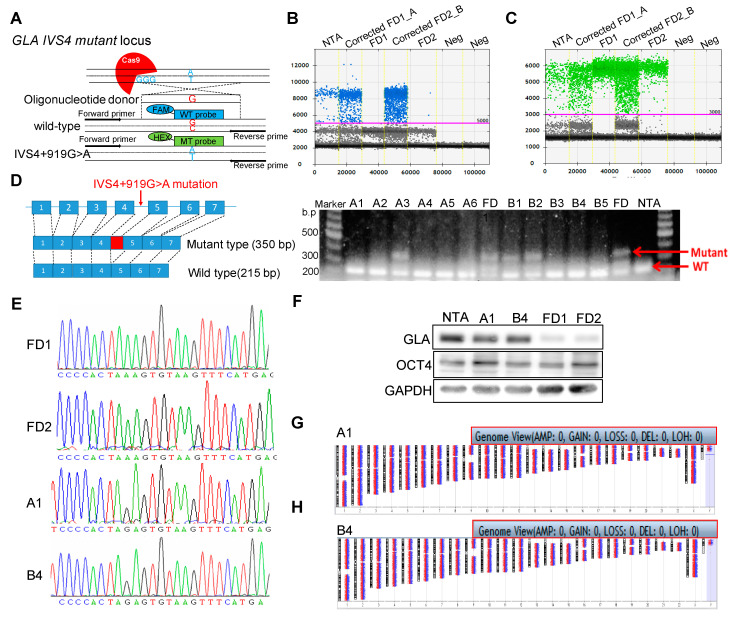
CRISPR/Cas9 corrected FD *GLA* IVS4 + 919G > A mutated induced pluripotent stem cells (iPSCs). (**A**) Schematic of guide RNA (gRNA) and donor template targeted region of the *GLA* locus of the patients with IVS4 + 919G > A mutation and probe design for homology-directed repair (HDR) genome-edited detection assay for droplet digital PCR (ddPCR)-based screening. Sensitive detection of *GLA* IVS4 + 919G > A mutant correction was performed using allelic discrimination edit detection assay by (**B**) 5-Carboxyfluorescein (FAM, blue) probe for Wild type (WT) allele and (**C**) Hex probe for mutant allele. (**D**) Reverse transcript PCR screening for GLA mutation of isolated clones after genetic correction by CRISPR/Cas9 and the single-stranded oligo donor (ssODN) template. (**E**) Sequencing results of selected corrected FABRY disease-iPSC (FD-iPSC) clones, A1 and B4. The light yellow box reveals mutant nucleotides substituted from A to G at IVS4 + 919 codon in FD-iPSCs. (**F**) The GLA protein levels of the corrected and uncorrected FD-iPSCs were evaluated with Western blot analysis. (**G**,**H**) Cytogenetic analyses of corrected FD-iPSCs, A1 and B4, demonstrated normal karyotypes individually. NTA is normal hiPSCs which served as control for normal *GLA* genome and pluripotency marker.

To understand the role of hotspot mutations of FD, we established iPSCs from two unrelated FD patients identified with a *GLA* IVS4 + 919G > A mutation (FD1 and FD2) and diagnosed as having hypertrophic cardiomyopathy, as well as one healthy adult control individual with no clinical manifestation (Table S1) [20]. We adopted CRISPR/Cas9-mediated homology-directed repair (HDR) genome editing technology to generate the isogenic control cells with correction of the mutation in these FD-iPSCs. Guide RNAs (gRNAs) targeting the *GLA* IVS4 + 919G > A mutation were designed and a single-strand DNA donor template (ssDNA) was inserted (Figure 1A). T7E1 assay confirmed the gRNA target site in the *GLA* locus (Figure S1A). The gRNA was constructed into a PX458 plasmid which contains GFP expression, and the transfection efficiency and enrichment of edited cells were determined by cell sorting. At 48 h post-nucleofection, the PX458-transfected cells carrying GFP were harvested for flow cytometry analysis (Figure S1B). We obtained approximately 30% transfection efficiency in the FD-iPSCs, which was demonstrated in a mixed population of cells with heterogeneous insertion–deletion (Indel) mutations with varying allelic editing frequencies. We applied droplet digital PCR (ddPCR)-based assays to distinguish between homo- and heterozygous edits in the clonal population by using a5-Carboxyfluorescein probe (FAM, blue) designed to detect the wild-type sequence (Figure 1B) and a Hex probe (green) designed to detect the mutated sequence as a reference (Figure 1C). Quantification of ddPCR results revealed that the corrected *GLA* IVS4 + 919 G > A mutation cell pools in A and B, from FD1 and FD2 individually, had high ratios of FAM-positive droplets, suggesting an abundance of HDR-edited alleles (Figure S1C). To isolate the HDR-edited cells, the pools of A and B cells were series diluted and expanded as single cell clones. We isolated clone A1 from the corrected FD1 pool A and clone B4 from the corrected FD2 pool B and validated their normal nucleotide length of the 215 bp splicing form, but not the aberrant 350 bp splicing form, in the *GLA* gene with the IVS4 + 919G > A mutation (Figure 1D). We also identified the correct nucleotide sequences at *GLA* IVS4 + 919 in chromatograms (Figure 1E) as the correct corrected FD for FD-iPSCs. ddPCR detection led to a strong FAM/Hex signal in corrected FD-A1 and B4 compared to the other heterozygous or FD-iPSC clones (Figure S1D). We also confirmed the restoration of GLA protein expression (Figure 1F) and GLA enzyme activity (Figure S2A) in corrected FD clones A1 and B4. The morphology and alkaline phosphatase activities were examined to determine the pluripotency of these corrected FD-iPSC lines (Figure S2B). Both A1 and B4 demonstrated the typical morphological characteristics of pluripotent stem cells and the expression of pluripotency markers (Figure S2C,D). The pluripotent ability was further determined by differentiating both corrected FD iPSCs into tridermal lineages (Figure S2F); there was no aberration in the chromosome by karyotype (Figure 1G,H). Taken together, the gene correction using CRISPR/Cas9 technology rescued GLA protein expression in FD-iPSCs with the IVS4 + 919G > A mutation.

### 2.2. Efficient Differentiation of Corrected FD-iPSCs into Functional Endothelial Cells

**Figure 2:**
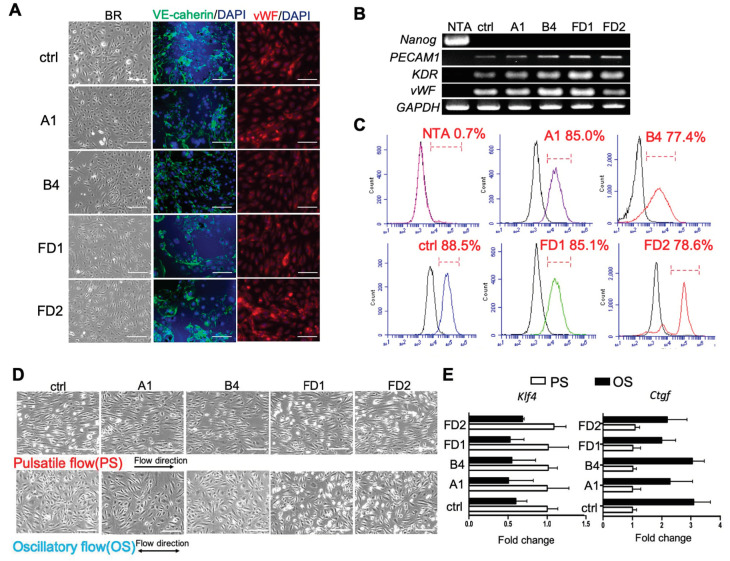
Characterization of the ECs differentiated from corrected FD-iPSCs and FD-iPSCs. (**A**) Characterizations of ECs of corrected FD-iPSCs and FD-iPSCs by phase contrast microscopy and immunostaining. The cells were stained with endothelium markers VE-cadherin and vWF. HUVECs were used as positive control (ctrl). The scale bar is 100 µm. n = 4 images from 4 biological replicates. (**B**) RT-PCR for endothelial markers (*PECAM1*, *KDF*, and *vWF*), and pluripotent marker (*Nanog*, negative control), for ECs after 30 days of differentiation. HUVECs were used as a control for normal *GLA* genome (ctrl). NTA is normal hiPSCs which served as control for pluripotency. *n* = 4 images from 4 biological replicates. (**C**) Histogram analyses of CD31 in corrected FD-ECs, FD-ECs, and HUVECs by FACS. *n* = 4 images from 4 biological replicates. (**D**) Morphological regulation of corrected FD-ECs and FD-ECs after 24 h of pulsatile shear stress (PS) and oscillatory shear stress (OS). The arrow indicates direction of flow. *n* = 4 images from 4 biological replicates. (**E**) Relative changes of *klf4* and *Ctgf* in corrected FD-ECs and FD-ECs after 24 h of PS and OS. The scale bar is 100 μm. The error bars represent SD.

To elucidate the mechanism underlying the FD-associated vascular endothelial dysfunction, the FD-iPSCs and corrected FD-iPSCs were differentiated into endothelial cells (ECs) using an established protocol [21]. After 14 days of differentiation, ECs were isolated using magnetic beads conjugated with an antibody against the surface CD31/PECAM-1. Immunostaining demonstrated that both FD-ECs and corrected FD-ECs expressed VE-cadherin and *von Willebrand Factor* (vWF) similar to those of HUVECs as control (ctrl) (Figure 2A). Quantitative RT-PCR analyses on Embryonic stem cells (ECs) obtained from differentiation of corrected FD-iPSCs and FD-iPSCs showed increases in endothelial markers such as *KDR*, *PECAM1*, and *vWF* (Figure 2B). Flow cytometry analyses showed that more than 80% of the differentiated cells (from FD and corrected FD-iPSCs) were CD31 positive, at a level similar to that of control (Figure 2C). These endothelial cells derived from iPSCs were used to study pathophysiological events in a patient-specific manner. We first characterized the mechanical regulation of these iPSC-ECs by subjecting them to atherosclerosis-protective (pulsatile shear, PS) and atherosclerosis-prone (oscillatory shear, OS) flow patterns in vitro [22] for 24 h to assess the changes of their morphology and gene expression. The results confirmed that PS (with large forward direction) caused these iPSCs-ECs to align in the direction of flow, while OS (with no forward direction) did not affect cell alignment (Figure 2D). We also observed that a key molecular marker of atheroprotection, Krüppel-like factor 4 (*KLF4)* [23], was induced when these ECs derived from iPSCs were subjected to PS. In contrast, the OS upregulated connective tissue growth factor (*CTGF*) [24], a potent vasoconstrictor known to be differentially regulated by flow patterns (Figure 2E). Together, these results demonstrated that both the FD-iPSCs and corrected FD-iPSCs can be efficiently differentiated into functional ECs.

### 2.3. Attenuations of GLA-Deficient Phenotypic Abnormalities in the Corrected FD-ECs

**Figure 3:**
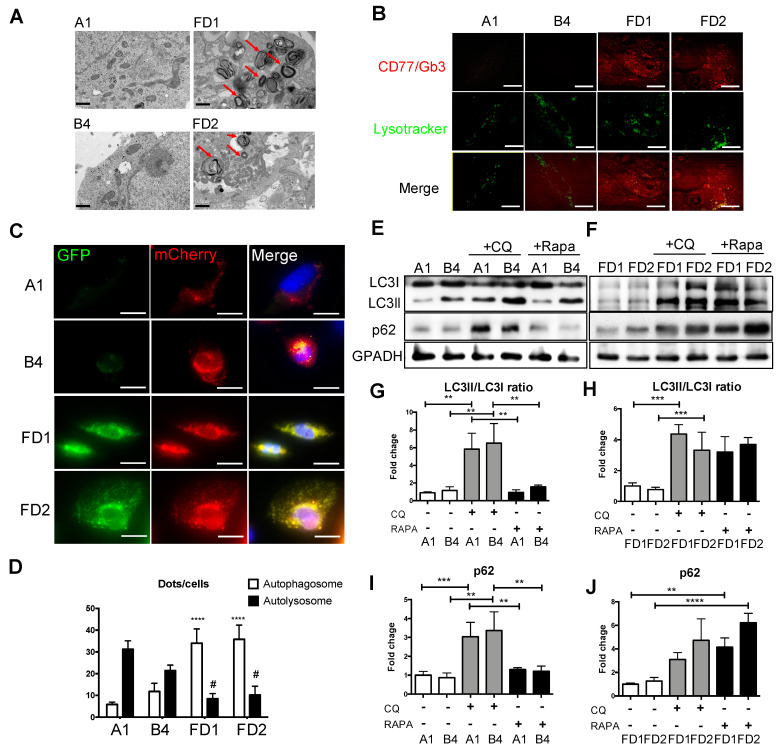
Attenuations of GLA-deficient phenotypic abnormalities in isogenic iPSCs-ECs. (**A**) Ultrastructure of corrected FD and FD-ECs. Arrowheads indicate multilayered lysosome structure. The scale bar is 0.5 µm. *n* = 4 images from 4 biological replicates. (**B**) Intracellular Gb3 was immunostained by CD77 in red plots, and lysosome was stained by lysotracker in green plots. The scale bar is 100 µm. *n* = 4 images from 4 biological replicates. (**C**) Images of iPSC-ECs from corrected FD and FD lines expressing tandem fluorescent mCherryGFP-LC3II (tf-LC3II). The scale bar is 100 µm. *n* = 30 images from 4 biological replicates. (**D**) Quantification of early and mature AVs in corrected FD and FD-iPSCs derived ECs expressing LC3II. * Compared to corrected FD in autophagosome; ^#^ compared to corrected FD in autolysosome. (**E**) Corrected FD and (**F**) FD-ECs were treated with 1uM chloroquine (CQ) or 200 nM rapamycin (Rapa) overnight, and protein levels of light chain 3 (LC3)-I and LC3II were evaluated by Western blot analysis. Quantification of (**G**,**H**) LC3II/LC3I ratio and (**I**,**J**) p62 expression levels (*n* = 3 images from 3 biological replicates). The scale bar is 100 μm. The error bars represent SD. Unpaired Student’s *t*-tests were used for statistical analysis (** *p* < 0.01; *** *p* < 0.005; **** *p* < 0.001; ^#^ *p* < 0.05).

To determine the genotype correction by CRISPR/Cas9 editing, we conducted real-time PCR analyses with specific primers for different *GLA* splicing forms. The results showed that the normal splicing form was expressed at higher levels (~5-fold) in the G genotypes than the A genotypes, and that the expression levels of the abnormal splicing form that included a stretch of intron showed the opposite pattern (Figure S3A,B). Our previous studies have shown that FD-ECs derived from iPSCs are impaired in Gb3 catabolism [17]. Consistent with these studies, we observed accumulation of Gb3 as a multilayered lysosomal structure along with the classic pathological phenotype of FD-ECs, and that such Gb3 accumulations were absent in corrected FD-ECs (Figure 3A). Similarly, immunofluorescence staining of Gb3 demonstrated significant cellular accumulation of Gb3 in FD-ECs, but not in corrected FD-ECs (Figure 3B). Since the Gb3 accumulation has been demonstrated to be an autophagy disorder, we further evaluated whether the autophagy impairment was rescued in corrected FD-ECs compared to FD-ECs. LC3-II and p62 are associated with autophagosome membranes which are readily degraded by lysosomal enzymes upon autophagosome-lysosome fusion [25]. The mCherry-GFP-LC3II construct was designed to have GFP quenched upon autophagosome-lysosome fusion, while mCherry remains stably expressed; early autophagic vacuoles (AVs) express both mCherry and GFP signals, whereas mature AVs express only the mCherry signal [26]. Quantification of mCherry and GFP AVs demonstrated that FD-ECs exhibited an increase in mCherry and a reduction in GFP compared to corrected FD-ECs (Figure 3C,D). To further demonstrate the correction of autophagic impairment caused by *GLA* IVS4 + 919G > A mutation, we evaluated the changes in the LC3ll/I and p62 levels in corrected FD-ECs (Figure 3E) and FD-ECs (Figure 3F) in the presence of the autophagy inhibitor chloroquine (CQ) and activator rapamycin (Rapa). CQ treatment caused a higher LC3II/LC3I ratio and p62 level in both corrected FD-ECs and FD-ECs. However, Rapa treatment increased the LC3II/LC3I ratio only in the FD-ECs, and not in corrected FD-ECs, indicating that the ability of autophagic flux was impaired in FD-ECs (Figure 3G–J). These results demonstrated that autophagic functions were rescued in the corrected FD-ECs. Impaired autophagy has been reported to block the reconditioning of organelles, including mitochondrial metabolism in cells, which triggered reactive oxygen species (ROS) production [27]. We found the mitochondria to be rounded and fragmented in FD-ECs, but not in the corrected FD-ECs (Figure S4A). Determination of intracellular ROS contents using Dichlorodihydrofluorescein diacetate (DCFDA) staining revealed that GLA correction or treatment with the potent antioxidant N-acetylcysteine (NAC) significantly decreased ROS levels in FD-ECs compared to the corrected FD-ECs (Figure S4B). In summary, we established that the corrected FD-ECs from patient-specific FD-ECs corrected the genetic mutation and reversed the autophagic impairment. This provides a valuable experimental platform to study the pathophysiological mechanism of FD-associated vascular endothelial dysfunction.

### 2.4. Enrichment of gene ontology (GO) Categories for Differentially Expressed Genes upon Correction of GLA IVS4 + 919G > A mutation

**Figure 4:**
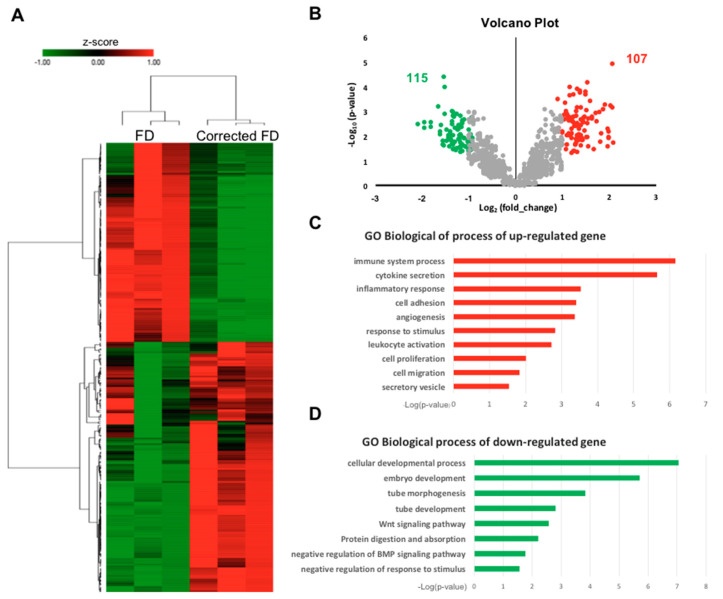
Differential expression analyses of corrected FD and FD-ECs derived from iPSCs. (**A**) Heatmap representing relative expression levels of the differential expression genes (DEGs) in corrected FD versus FD-ECs with fold change ≥ 2 up or down on average in both lines. Samples (in columns) and genes (in rows) are clustered by similarity. Shades of red represent upregulation, shades of green represent downregulation. Z score refers to the expression level of the indicated genes. (**B**) Volcano plot reveals 107 DEGs in red plot with fold of induction ≥ 2 and 115 DEGs in green plot with fold of reduction ≤ 2 in FD-ECs versus isogenic control. (**C**) Gene ontology (GO) pathway analysis of 107 DEGs are upregulated in FD-ECs versus isogenic control. (**D**) In gene ontology (GO) pathway analysis, 115 DEGs are downregulated in FD versus isogenic control ECs. The *p*-values were calculated with Benjamini-Hochberg adjustment.

To explore the molecular pathways modulated by the *GLA* IVS4 + 919G > A mutation, we performed microarray gene profiles for corrected FD and FD-ECs. Unsupervised hierarchical clustering of the microarray data showed distinct segregation of gene profiles between FD-ECs and the corrected FD-ECs, indicating that the *GLA* IVS4 + 919 G > A allele had a significant impact on the transcriptome (Figure 4A). Data analyses identified 107 upregulated genes and 115 downregulated genes in the FD-EC dataset, with a *p*-value less than 0.05, when compared with corrected FD-ECs (Figure 4B). To decipher the regulatory pathways, gene ontology (GO) term analysis was performed. We identified that pathways in the categories of regulation of cytokine secretion, angiogenesis, and inflammatory response were enriched in the upregulated genes (Figure 4C), and that the categories of tube development, Wnt signaling, and protein digestion were enriched in the downregulated genes (Figure 4D) in FD-ECs. These results suggest that the Gb3 accumulated by the *GLA* mutation in IVS4-919G > A is positively correlated to the inflammatory signaling pathways, but inversely correlated to the developmental signaling.

### 2.5. Validation of GLA Mutation Involved Inflammatory Response in FD and Corrected FD-ECs

**Figure 5:**
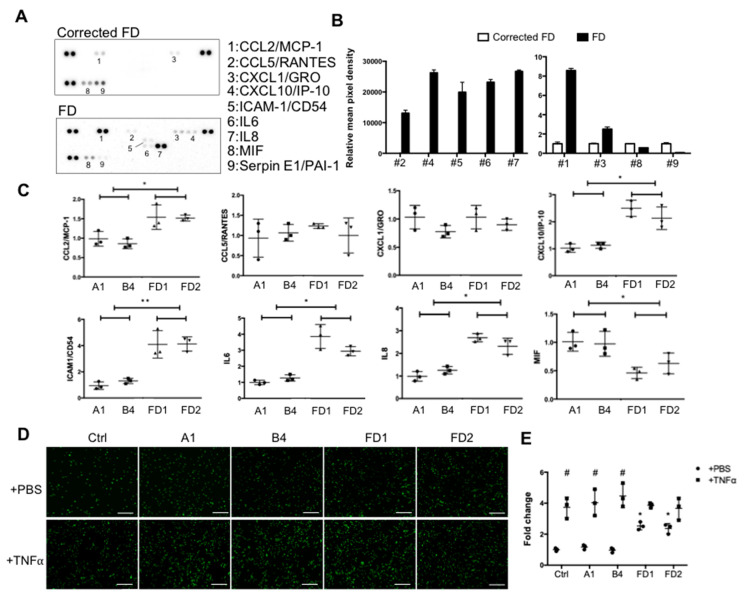
Cytokine releases and inflammatory status in corrected FD and FD-ECs. (**A**) Cytokine levels in conditioned media of corrected FD and FD-ECs were measured using a human cytokine Multi-Analyst ELISArray Kit and (**B**) normalized to the conditioned media of isogenic control. (**C**) Expression levels of pro-inflammatory genes validated by qRT-PCR (*n* = 3 images from 3 biological replicates). (**D**) Representative images for monocyte (THP-1) adhesion in corrected FD and FD iPSC-ECs. HUVECs were used as a control (ctrl). (**E**) Quantification of THP-1 adhesion of experiments (*n* = 3 images from 3 biological replicates). * Compared to corrected FD individually. ^#^ Compared to PBS in TNFα treatment. The error bars represent SD. One-way ANOVA was used for statistical analysis (* *p* < 0.05; ** *p* < 0.01; ^#^ *p* < 0.05).

**Figure 6:**
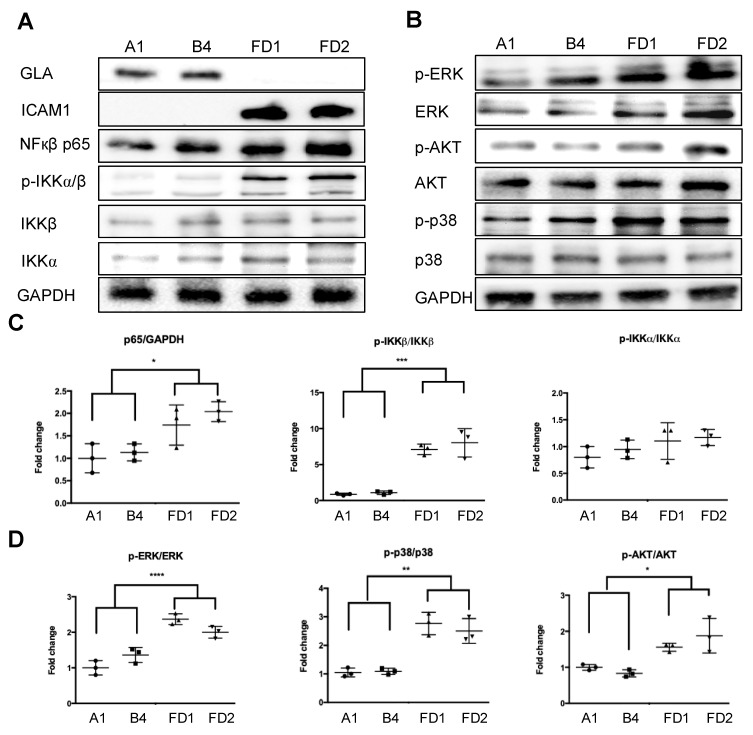
(**A**) Corrected FD-ECs attenuate NF-κB and MAPK signaling pathway in comparison to FD-ECs. Expression levels of GLA, ICAM1, and p65, as well as the phosphorylation levels of IKKα and IKKβ, examined by Western blotting. (**B**) Phosphorylation of ERK, AKT, and p38 in the MAPK signaling pathway examined by Western blotting. (**C**) Quantitative analyses of p65 expression and IKKα and IKKβ phosphorylation in corrected FD-ECs compared to FD-ECs (*n* = 3). (**D**) Quantitative analyses of phosphorylation of ERK, AKT, and p38 in corrected FD-ECs compared to FD-ECs (*n* = 3). Error bars represent SD. One-way ANOVA was used for statistical analysis (* *p* < 0.05; ** *p* < 0.01; *** *p* < 0.005; **** *p* < 0.001).

To further validate and investigate the biological relevance of these differentially expressed genes, we focused on studying their roles in the inflammatory response and cytokine secretion that contribute to Fabry cardiovascular pathogenesis [28]. A human cytokine Multi-Analyte ELIiSArray Kit (R&D) was used to examine the levels of 42 cytokines related to immunity and inflammation. Basal conditioned media from FD-ECs and corrected FD-ECs were collected and subjected to the cytokine array. The results demonstrated that the correction of *GLA* IVS4 + 919G > A mutation in corrected FD-ECs significantly reduced the secretion of pro-inflammatory cytokines CCL2/MCP-1, CCL5/RANTES, CXCL1/GRO, CXCL10/IP-10, ICAM-1/CD54, IL6, and IL8 when compared with FD-ECs. Conversely, the secretion of MIF and Serpin E1/PAI1 was significantly lower in the FD-ECs (Figure 5A,B). The RNA levels of the these proinflammatory cytokines were examined by real-time PCR, and the results validated the cytokine array analysis with the exception of *CCL5/Rantes* and *CXCL1/GRO,* which did not reveal significant difference between corrected FD-ECs and FD-ECs. We further compared the functional consequences of the corrected FD-ECs and FD-ECs by assaying the monocyte recruitment. The results demonstrated that correction of *GLA* IVS4 + 919G > A mutation in corrected FD-ECs significantly reduced the monocyte attachment in comparison to the FD-ECs (Figure 5D,E). Western blotting demonstrated that the expression levels of p65 and ICAM1(Figure 6A) and the phosphorylation levels of IKKα, IKKβ, ERK, AKT, and p38 (Figure 6B–D) were significantly higher in FD-ECs when compared with corrected FD-ECs. These findings indicate that *GLA* IVS4 + 919G > A mutation impacted on the inflammation-related NF-κB and MAPK signaling pathways.

## 3. Discussion

In the Fabry disease iPSC-based models, iPSCs derived from healthy individuals with different genetic backgrounds are usually used as controls for patient-derived iPSCs [29,30]. It is essential, however, to generate isogenic iPSC lines with the key mutant gene as the sole variable, thus allowing for identification of the actual pathological phenotypes, without interferences resulting from different genetic or epigenetic backgrounds. The novel and important feature of this study is that we have used CRISPR-based gene editing to permanently correct a pathologic mutation in FD patient-specific iPSCs. We have experimentally corrected the *GLA* mutation in IVS4 + 919G > A that causes a late-onset FD cardiac-variant pathology. Although previous studies reported that FD cardiomyopathy can be recaptured with CRISPR/Cas9-edited *GLA* knockout in embryonic stem cells [31], one of them established the isogenic control [32] to provide definitive evidence in identifying the role of *GLA* IVS4 + 919G > A mutation in regulating FD cardiomyopathy and elucidating the mechanisms involved.

Homologous recombination (HR)-mediated precise gene repair has been reported to be particularly difficult in human pluripotent stem cells. A few publications have described the combination of engineered nucleases with a donor vector as a therapeutic strategy for gene modification, such as hemophilia A, sickle cell disease [33], and Duchenne muscular dystrophy (DMD) [34]. These results revealed that the single-base substitutions or deletions often occur at frequencies below 1%. Moreover, most of the groups adopted selection makers to enhance the substitution frequency. It is to be noted that after selection, some sequences may increase the genome instability in the corrected iPSCs and interfere with the expression of the corrected gene [35]. Therefore, it is critical to select and isolate cells effectively to ensure optimal modeling of the disease. We applied the method of droplet digital PCR (ddPCR)-based detection [36] to efficiently correct *GLA* IVS4 + 919G > A mutation in FD-iPSCs. Compared to the conventional mutation screening method, ddPCR requires very little genomic DNA samples to identify the single-cell-derived clones with a single mutated allele (Figure 1). CRISPR/Cas9-corrected *GLA* IVS4 + 919G > A mutation clones possess identical genetic background except for *GLA*, and served as an ideal model to study the pathogenic effect of *GLA* IVS4 + 919G > A mutation during endothelial development (Figure 2) and clearance of the mitochondrial dysfunction though reversing autophagic flux (Figure 3). Therefore, CRISPR/Cas9-corrected-derived ECs are particularly valuable in revealing the phenotype differences that are specific to the *GLA* IVS4 + 919G > A mutation. Contrary to the view that *GLA* plays only a structural role in glycolipid catabolism, the microarray sequencing of the transcriptomes of *GLA* IVS4 + 919G > A mutated ECs at development from iPSCs revealed changes in transcription as compared to the control, such as interference of tube formation and stimulation of inflammatory cytokine secretion (Figure 4 and Figure 5). Glycosphingolipid deposits significantly affect the functions of vascular endothelium by inducing the degradation of the membrane Ca2^+^-activated K^+^ channel (KCa3.1) that causes dysregulation of endothelium-dependent relaxation [37]. Autophagy flux impairment caused by lysosomal failure in FD disease has been considered as a major factor in producing ROS and contributing to pro-inflammatory cascade events [38]. Although inflammation protects all multicellular animals against exogenous pathogens [39], it is also a major pathogenic cofactor of many chronic human diseases when the dysregulated inflammatory response persists [40]. Not only lysosomal storage diseases (LSDs) such as FD [38,41], but also neurodegenerative diseases such as Alzheimer’s [42] and Parkinson’s disease [43], are associated with an inflammation-related autophagosome–lysosome fusion impairment for abnormal protein accumulation [44]. In recent years, it has become clear that inflammation and activation of the innate immune system are a general response in Fabry disease and are primarily caused by Gb3 accumulation. The massive accumulation of intracellular Gb3 enhanced the production of reactive oxygen species (ROS) and the expression of cell adhesion molecules in ECs [45,46]. On the other hand, enzyme replacement therapy is an effective treatment strategy for FD patients, especially intracellular deposits of Gb3 in the endothelial cells, which eliminated oxidative stress and inflammation to retard the disease progress [47]. In FD with *GLA* IVS4 + 919G > A mutated patients, their left ventricular mass index is correlated with changes in IL-6 and MCP-1, indicating a potential use of these cytokines as pro-inflammatory agents and biomarkers [48]. We found changes in both the abundance and phosphorylation status of proteins within the p38 MAPK pathway in CRISPR/Cas9-edited corrected FD-ECs (Figure 6). Transforming growth factor-beta (TGF-β) which regulated p38 MAPK by hyperosmolarity, oxidative stress, and/or inflammatory cytokines to lead to the phosphorylation of downstream targets and activated the nuclear transcription factor-induced apoptosis response [49], showed differences in its protein abundance in podocytes and aortic endothelial cells of Fabry disease patients with nephropathy [50,51]. Activation of TGF-β signaling characterized by endothelial-to-mesenchymal transition (EMT), i.e., loss of endothelial and gain of mesenchymal phenotype, is an important mechanism in the pathogenesis of fibrotic disorders [52]. Gb3 and Lyso-Gb3 strongly induced epithelial–mesenchymal transition (EMT) in human proximal tubule HK2 cells, contributing to the development of renal fibrosis through the cell-specific induction of EMT in FD [53]. These findings suggest that the glycosphingolipid metabolites accumulated in FD are biologically active and contribute to triggering EMT that resulted in the progression of cardiomyopathy.

## 4. Materials and Methods

### 4.1. Genome Editing of FD GLA IVS4 + 919G > A iPSCs

Conversion of A to G in the No. 919 nucleotide in intron 4 of the *GLA* gene with single-stranded oligo donor (ssODN) was performed in accordance with the following procedure. Two independent FD patient iPSC lines that have been established previously [20] and maintained on Geltrex^™^ (Thermo Fisher Scientific, Waltham, MA, USA)-coated 60 mm dishes in mTESR1 medium (StemCell Technologies, Vancouver, BC, Canada) were used for transfection. Before transfection, the cells were incubated with 10 μM ROCK Inhibitor (StemCell Technologies, BC, Canada) for 2 h. Then the cells were dissociated into single cells by TrypLE^™^ Select (Life Technologies, CA, USA) and counted. pSpCas9(BB)-2A-GFP (PX458) was a gift from Feng Zhang (Addgene plasmid #48138; http://n2t.net/addgene:48138, accessed on 1 January 2021; RRID: Addgene_48138), and corrected plasmid was made as described [54]. gRNA (5’-ACAAATACTTCCAAATAGTGTGG-3’) was designed and constructed with the PX458 plasmid presented on the website of the Zhang Lab, MIT (http://crispr.mit.edu/, accessed on 1 January 2021) targeting the mutated site. A 90 nt single-stranded oligodeoxynucleotide (ssODN) was synthesized by ToolGen, Inc. Subsequently, 6 μg of corrected plasmid and 1μL of 100 μM ssODN were mixed with 1.6 × 10^6^ FD-iPSCs using 100 μL of Human Stem Cell Nucleofector Kit 2 (Lonza) and nucleofected with Nucleofector II under program B016. Cells were plated on Matrigel-coated 6-well plates in mTeSR1 containing 10 μM Y27632 for 24 h. The expression of restored enhanced green fluorescent protein (EGFP) was measured by fluorescence microscopy and flow cytometry, and the percentages of GFP-positive cells were analyzed and sorted by FACS-Calibur^™^ (BD Biosciences) at 48 h after transfection. Another 3 days later, cells were detached with TrypLE^™^ Select and counted. One thousand cells were replated on mouse embryonic fibroblast feeder cells in embryonic stem cell (ES) medium supplemented with 10 μM Y27632 for 24 h and changed to ES medium without Y27632 every day until clone picking.

### 4.2. Array-Based Comparative Genomic Hybridization (Array CGH) 

Here, we performed array-based comparative genomic hybridization to demonstrate that there was no chromosome aberration after the CRISPR/Cas9-mediated genome-editing intervention. The genomic DNA of iPSCs with a low passage number (4th–7th passage) was isolated and intermittently sonicated using a Digital Sonifier 450 sonicator probe (Branson Ultrasonics, Danbury, CT, USA). DNA samples were amplified using the GenomePlex WGA kit (Thermo Fisher Scientific, Waltham, MA, USA). A Genomic DNA ULS Labeling Kit (Agilent) was used to label the amplified DNA with either Cy3 or Cy5. As recommended by Agilent, 2.0–2.5 g of amplified DNA were used as the input starting material for each labeling reaction. Scanning and image analysis were conducted according to the Agilent Oligonucleotide Array-based CGH for Genomic DNA Analysis Protocol (version 4.0). Microarrays were scanned using an Agilent G2565BA DNA Microarray Scanner (Agilent). Agilent Feature Extraction software (v9.1.3) was used to extract data from raw microarray image files. Agilent CGH Analytics software (v3.4) was used to visualize, detect, and analyze the aberration patterns from CGH microarray profiles.

### 4.3. Droplet Digital PCR (ddPCR)

The droplet digital PCR was carried out following published protocols [55]. Briefly, sequence-specific PCR primers and probes were designed (Table S2). The extracted genomic DNA was used for ddPCR. The assay consisted of the following components (final concentrations in 20 µL total reaction volume): ddPCR SuperMix for Probes (no dUTP) (1×), forward primer (900 nM), reverse primer (900 nM), reference probe (HDR-insensitive probe, Hex, 250 nM), HDR-sensitive probe (different fluorophore than reference; FAM, 250 nM), nuclease-free water, and ~40 ng gDNA was used as template. All primers and probes were designed using Primer3 plus (http://primer3plus.com, accessed on 1 January 2021) from Eurofns (Eurofns Genomics, Louisville, KY, USA). All ddPCR assays were analyzed using the QX100 droplet reader and Quantasoft software version 1.7.4 (Bio-Rad). Genome editing was calculated according to the ratio based on the concentrations of events per µL, and it was used for the calculation of gene-editing frequency.

### 4.4. Differentiation of iPSCs to Endothelial Cells (ECs)

We differentiated iPSCs to ECs according to the established monolayer EC differentiation protocol with a minor modification. Briefly, iPSC clumps were seeded into a six-well plate coated with Geltrex™ (Thermo Fisher Scientific, Waltham, MA, USA) and incubated with STEMdiff APEL^™^ endoderm differentiation medium (StemCell Technologies, Vancouver, BC, Canada) containing activin A (25 ng/mL; Cayman, Ann Arbor, MI, USA), BMP4 (30 ng/mL; Cayman), CHIR (1.5 mM; Cayman), and vascular endothelial growth factor (VEGF; 50 ng/mL; R&D Systems, Minneapolis, MN, USA). After 3 days, the medium was changed to STEMdiff APEL^™^ containing VEGF (50 ng/mL) and SB431542 (10 nM; TOCRIS, Bristol, UK) and again at days 10 and 13. ECs were isolated on day 14 using CD31-conjugated magnet beads (StemCell Technologies, Vancouver, BC, Canada). The isolated ECs were further cultured in endothelial cell growth medium-2 (EGM-2) (Lonza) complemented with 5% fetal bovine serum (FBS).

### 4.5. Shear Stress Experiment

To apply mechanical shear forces on HUVECs and iPSC-derived ECs, an in vitro circulating flow chamber system was used to impose fluid shear stress on the ECs [32]. The HUVECs cultured on collagen I-coated slides were exposed to PS (12 ± 4 dyn/cm^2^, 1 Hz) or OS (0.5 ± 4 dyn/cm^2^, 1 Hz) for 24 h.

### 4.6. GLA Enzyme Activity

Cells were washed twice with 1X PBS and were lysed in 60 μL lysis buffer (27 mM sodium citrate, 46 mM sodium phosphate dibasic, 0.5% Triton X-100). Ten microliters of cell lysate were added to 50 μL assay buffer containing 6 mM 4-methylumbelliferyl-α-d-galactopyranoside and 117 mM N-acetyl-D-galactosamine and incubated at 37 °C for 1 h. The 4-methylumbelliferone dissolved in methanol was used as a standard, ranging from 0.15 μM to 5000 μM. Thereafter, 70 μL glycine carbonate solution (pH 10.8) were then added to stop the reaction and fluorescence was detected by a microplate reader (em/ex = 365/448 nm). The enzyme activity was normalized by the protein concentration of the cell lysate.

### 4.7. Immunofluorescence

Immunofluorescence staining was performed as described previously [56] with some modifications. Cells were fixed with 1% paraformaldehyde (PFA) (Sigma-Aldrich) solution at room temperature for 15 min and permeabilized with 0.1% Triton X-100 (Merck, Darmstadt, Germany) for 10 min. After several washes with 1 × PBS, the fixed cells were blocked using 3% bovine serum albumin (BSA; Bovogen Biologicals, VIC, Australia) and 5% FBS, and subsequently incubated with indicated monoclonal antibodies (1:100) at 4 °C overnight. Cells were washed thrice with PBS and incubated with the cyanine 3 (Cy3)-conjugated goat anti-mouse IgG or FITC-conjugated goat anti-rabbit IgG secondary antibody (Thermo Fisher Scientific, Waltham, MA, USA) at room temperature for 1 h. Samples were counterstained with 100 μL DAPI. Finally, cells were mounted and observed using a fluorescent or FV10i confocal microscope (Olympus, Center Valley, PA, USA).

### 4.8. Quantitative Real-Time PCR (qRT-PCR)

Total RNA was prepared from cells or tissues using Trizol reagent according to the manufacturer’s protocol (Invitrogen). qRT-PCR of mRNAs was reverse-transcribed using the Superscript III first-strand synthesis system for RT-PCR (Invitrogen). qRT-PCR reactions of resulting cDNAs were performed on an ABI 7900HT (Applied Biosystems, Carlsbad, CA, USA). The appropriate primer sets are listed in Table S2.

### 4.9. Immunoblotting

Total proteins were separated through gradient sodium dodecyl sulfate polyacrylamide gel electrophoresis (SDS-PAGE) and transferred onto a polyvinylidene difluoride (PVDF) membrane. After blocking with 5% skim milk at room temperature for 1 h, the membrane was hybridized with primary antibodies in Tris-buffered saline Tween20 (TBST) at 4 °C overnight, followed by incubation with horseradish peroxidase-conjugated secondary antibodies at room temperature for 1 h. The immunoblot was developed using an enhanced chemiluminescence system (EMD Millipore, Darmstadt, Germany) and detected using an X-ray film (Fujifilm, Tokyo, Japan). The antibodies used are listed in Table S3.

### 4.10. Transmission Electron Microscopy

The morphology of differentiated endothelial cells was characterized using a transmission electron microscope (TEM, JEM-2000EXII, JEOL, Japan). The endothelial cells covered a 400-mesh carbon-coated copper TEM grid. After 15 min, the grid was tapped with filter paper to remove the excess water, followed by staining with 1% phosphotungstic acid (P4006, Sigma, St. Louis, USA) for 20 min. The samples were allowed to air dry for 24 h and then observed under TEM.

### 4.11. Autophagy Flux Sensor

Autophagic flux was monitored using the Premo™ Autophagy Tandem Sensor RFP-GFP-LC3II Kit (Thermo Fisher Scientific, Grand Island, NY, USA) following the manufacturer’s instructions. iPSC-derived ECs were treated with 10 μL of BacMam reagents containing the RFP-GFP-LC3II construct for a 12-h incubation followed by being washed with PBS and mounted with ProLong Gold Antifade Mountant (Thermo Fisher Scientific, Waltham, MA, USA). DAPI staining was used to label cell nuclei. Images were analyzed for green fluorescence (LC3II positive autophagosome) and red fluorescence (autophagolysosome formation) using a Zeiss inverted fluorescence microscope. More than 30 individual cells images were scored over four independent experiments.

### 4.12. Microarray Analysis and Bioinformatics

Total RNA was isolated using a standard Trizol protocol (Life Technologies, Bethesda, MD) (Chomczynski and Sacchi 2006) and the Qiagen RNAeasy (Qiagen, Valencia, CA, USA) column for purification. RNA purity and quality were measured by a UV spectrophotometer and an Agilent 2100 Bioanalyzer (Agilent Technologies, Foster City, CA, USA), and the RNA integrity number value was required to be > 8 for each RNA sample. Ten to fifteen micrograms of total RNA reverse transcribed from each sample was used in each cycle of microarray analysis. Affymetrix HG U133 Plus 2.0 microarrays containing 54,675 probe sets for > 47,000 transcripts and variants, including 38,500 human genes, were used. A typical probe set contains 25-mer oligonucleotide pairs (a perfect match and a mismatch control). Some genes were measured by multiple probe sets. Sample labeling, hybridization, and staining for microarray analysis were carried out with an Affymetrix standard protocol. The differentially expressed genes (≥2-fold changes) between control and Fabry sample were isolated. A heat map was visualized by MultiExperiment Viewer (MeV) downloaded from the TM4 microarray software suite (http://www.tm4.org/, accessed on 1 January 2021). The color presents the normalized expression value of log2 (fold change); red indicates upregulated genes, and green indicates the downregulated genes. Outputs were also visualized using the volcano plot function. A filtering approach to identify differentially abundant genes was also applied by first calculating the difference in expression of each Fabry sample compared to the mean of all the control samples in that group. Genes with ≥ 1x log2 fold change for both Fabry lines individually and an adjusted *p*-value < 0.05 with Benjamini–Hochberg adjustment using all samples (one-sample *t*-test; µ = 0) were selected. Each sample was considered as an independent replicate. The lower *p*-value used for filtering was used to reduce the candidates to a sensible number. Genes with more than one missing value in the more highly expressed group were discounted, after inspection. Classification of gene annotation was performed using the Gene Ontology Database (http://geneontology.org/, accessed on 1 January 2021). Z scores were calculated following the formula ($=\frac{\chi -\mu }{\sigma }$, μ=mean and σ=standard deviation) to indicate how far from the mean a data set is.

### 4.13. Cytokine Array

Human cytokine Array PlanA Kits (R&D systems, # ARY005) were used to measure the 36 cytokines, chemokines, and acute phase proteins as induction was revealed in the supernatants of conditioned media of corrected FD and FD-ECs after 48 h of culture. The arrays were performed according to the manufacturer’s instructions. The absorbance levels of the cytokines were measured by chemiluminescence which was detected in the same manner as a western blot.

### 4.14. Monocyte Adhesion Assay

THP-1 monocyte cells were maintained in RPMI-1640 medium containing 10% FBS. To assess the binding of THP-1 cells to ECs, the THP-1 cells were labeled with CellTracker™ Green CMFDA Dye (Thermo Fisher Scientific, Waltham, MA, USA) and then incubated with the iPSC-derived ECs for 30 min. The unattached THP-1 cells were washed away with endothelial growth medium and the number of attached cells was counted using fluorescence microscopy.

### 4.15. Statistical Analysis

Data are expressed as the mean ± standard derivation (SD) and were analyzed using GraphPad Prism 5.03 software with a two-tailed Student’s *t*-test for two groups or one-way ANOVA with Tukey’s post hoc test. Statistical significance of the observed changes was assumed at *p* < 0.05. A more detailed description of the methods used in this study can be found in the Supplemental Information.

## 5. Conclusions

We successfully established and studied isogenic control cells with the same genetic background as that in FD, with the correction of the *GLA* IVS4 + 919G > A mutation. Our study demonstrated that diseased FD-ECs can be restored via gene correction, providing the in vitro proof-of-concept evidence to support the mutation-repairing strategy with CRISPR/Cas9-mediated gene editing. The mutation-corrected FD-ECs are similar to healthy endothelial cells and have potential value for EC regeneration without the concerns of immunological rejection. Our model system could be used to provide great promise for the development of novel strategies and optimize in vivo gene therapy approaches for the prevention of and intervention in the adverse effects of FD-associated vasculopathy.

## Data Availability

The data presented in this study are available in this article and Supplemental Material.

## Supplementary Materials

Supplementary Materials can be found at https://www.mdpi.com/1422-0067/22/5/2381/s1, Figure S1: The effects of CRISPR/Cas9 correction in FD-iPSCs, Figure S2: Characterization of FD-iPSCs and corrected FD-iPSCs, Figure S3: Genotypes of FD-iPSCs and corrected FD-iPSCs, Figure S4: Phenotypes of FD-iPSCs and corrected FD-iPSCs, Table S1: Summary of healthy controls and patients’ cell lines, and Table S2: Sequences of the primers used for probe, RT-PCR, and qPCR.

## Abbreviations

LSD	Lysosomal storage disease
FD	Fabry disease
Gb3	Globotriaosylceramide
Gal A	galactosidase A
ERT	Enzyme replacement therapy
CRISPR	Clustered regularly interspaced short palindromic repeats
sgRNA	Single-guide RNA
Cas9	CRISPR-associated protein
ddPCR	Droplet digital PCR
ESCs	Embryonic stem cells
iPSCs	Induced pluripotent stem cells
CMs	Cardiomyocytes
ECs	Endothelial cells
HUVECs	Human umbilical vein endothelial cells
MVEs	Multivesicular endosomes
AVs	Autophagic vacuoles
ROS	Reactive oxygen species
EMT	Endothelial-to-mesenchymal transition
