# Supplementary material for: Reversal of the Inflammatory Responses in Fabry Patient iPSC-Derived Cardiovascular Endothelial Cells by CRISPR/Cas9-Corrected Mutation
Source: Int J Mol Sci. 2021 Feb 27;22(5):2381. doi: 10.3390/ijms22052381 (PMC7956852; doi:10.3390/ijms22052381)
Supplement: Supplementary file 1 [file ijms-22-02381-s001.pdf]

Supplementary Figure and Table

Supple Figure 1.

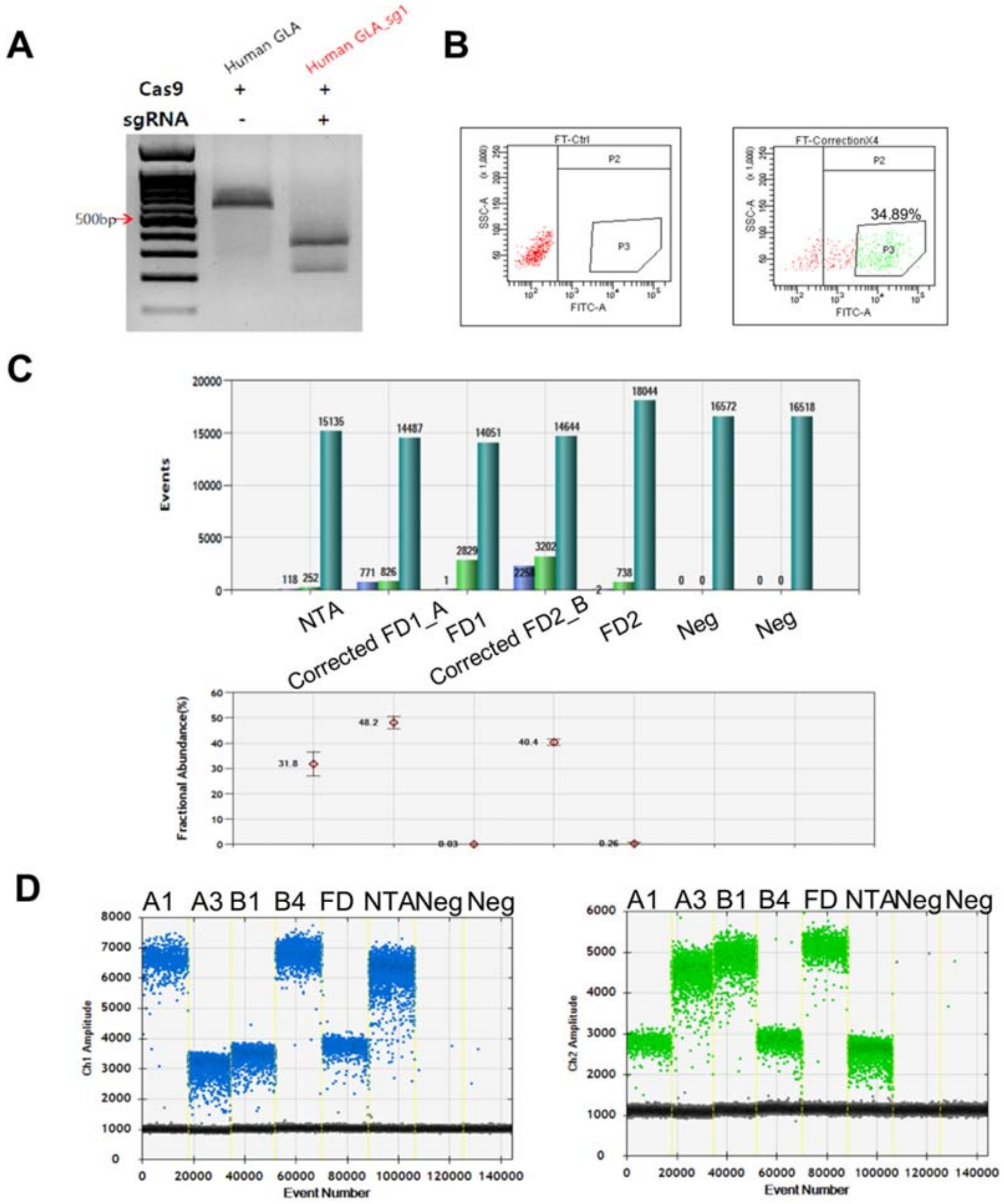

Supplementary Figure 1. (A) Genomic DNA of PX458-gRNA transfected HEK293T cells was extracted and the region spanning the gRNA target sites was PCR amplified using on-target primer pairs (arrows), giving PCR products of 544 bp (uncut) and 211 and 333 bp (cut). (B) Quantification of activity of targeted CRISPR/Cas9 using flow cytometry analysis. (C) Quantification of corrected and mutant cell number by ddPCR detection assay.

(D) ddPCR analysis of selected clones from corrected FD pools A and B.

Supple Figure 2.

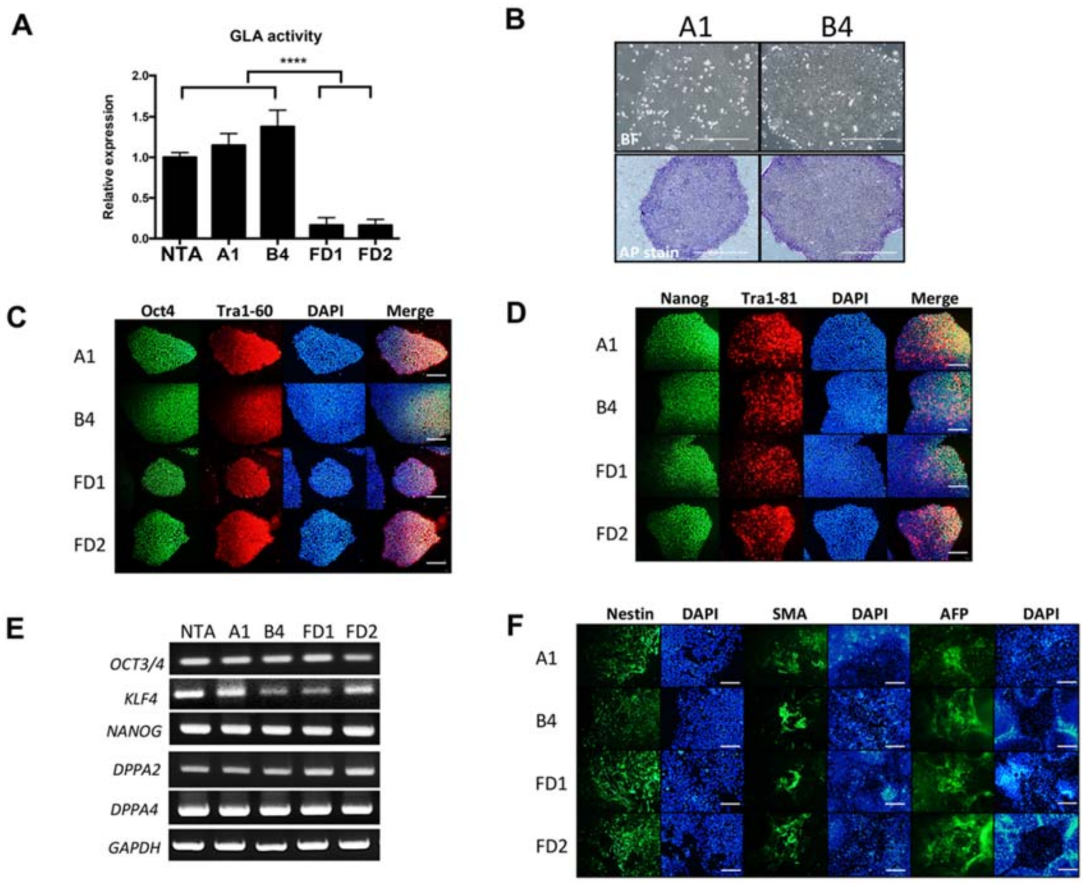

Supplementary Figure 2. (A) GLA enzyme activity of isognenic ctrl rescued from FD-iPSCs, which were compared to NTA, normal hiPSCs ctrl. (B) Morphology and alkaline phosphatase activity of isogenic ctrl-iPSCs. The scale bar is 100 μm. (C, D) Immunofluorescence analysis demonstrated the protein expression of pluripotency markers [(C) NANOG, Octamer-binding transcription factor 4 (OCT4), TRA-1–60, (D) Nanog and TRA-1–81] in isogenic Ctrl-iPSCs sublines (B1 and A4). Nuclei were counterstained with DAPI staining. The scale bars is 100 μm. (E) Reverse transcription polymerase chain reaction (RT-PCR) analysis indicated the expression pattern of embryonic stem cell-like genes in isogenic Ctrl-iPSC lines. NTA is normal hiPSCs from a health donor serving as a positive control. (F) *In vitro* three-layer differentiation of isogenic Ctrl-iPSCs in specific culture media resulted in subpopulations of cells that were immunoreactive for mesodermal smooth muscle actin (SMA), ectodermal Nestin, and endodermal α-fetoprotein (AFP). The scale bars is 100 μm. FD1, FD-iPSC1; FD2, FD-iPSC2; n = 4 images from 4 biological replicates.

Supple Figure 3.

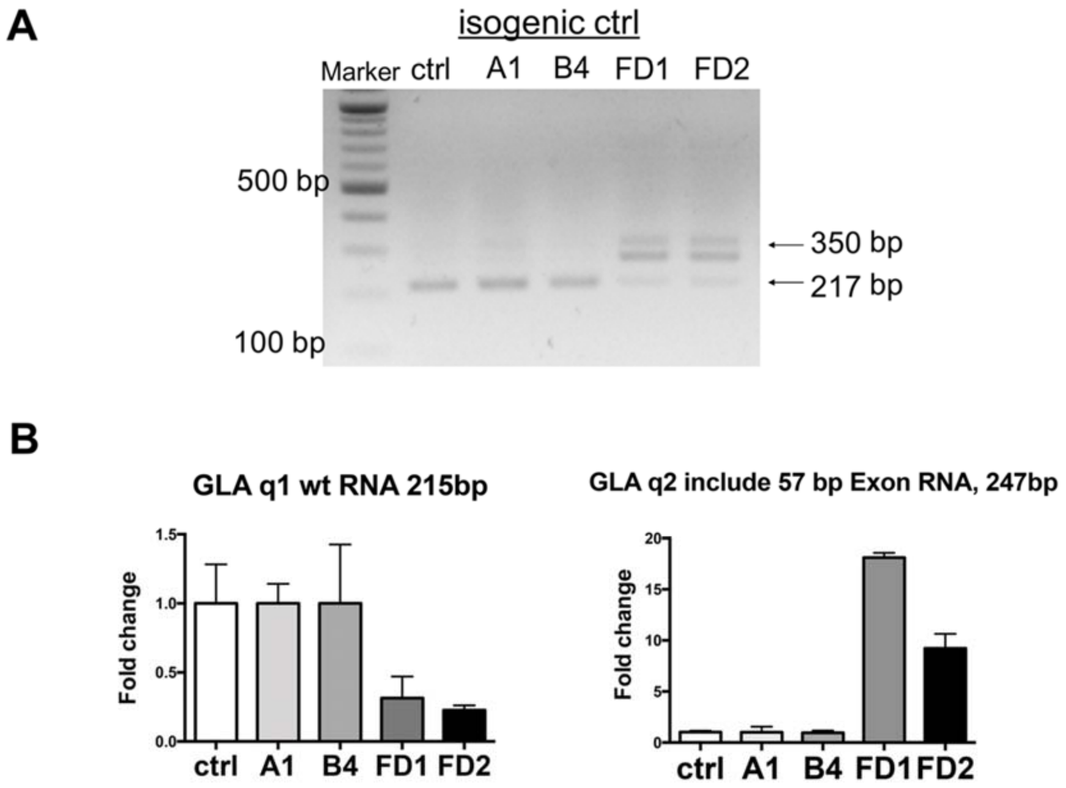

Supplementary Figure 3. (A) cDNA from isogenic ctrl and FD-ECs validated the different genotypes. Marker represents the 100-bp DNA ladder. HUVECs served as control (ctrl). (B) RT-PCR with specific primers (GLAqF, GLAq1R for q1 (wild type RNA) and GLAq2R for q2 (include 57bp exon RNA)) for different *GLA* splicing forms in different genotype cells.

Supple Figure 4.

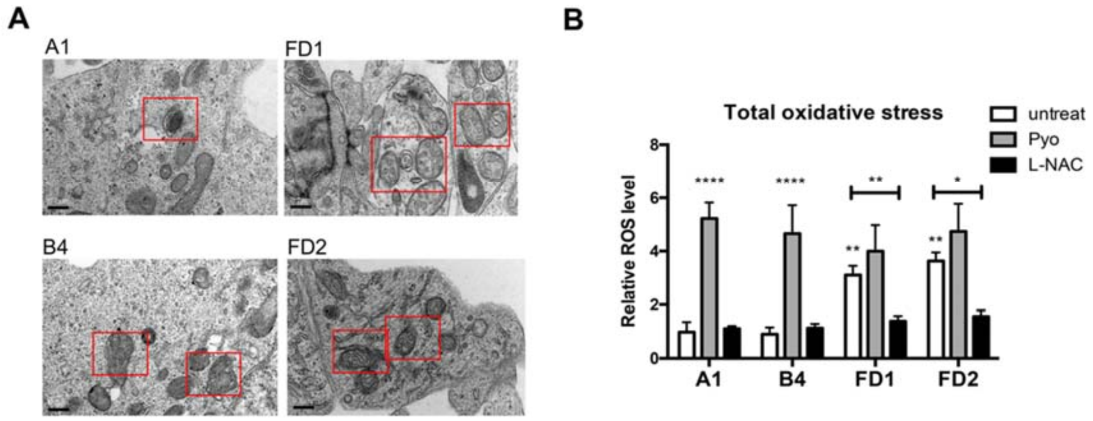

Supplementary Figure 4. (A) Transmission electron microscopy (TEM) of isogenic ctrl and FD-ECs revealed significant accumulation of intracytoplasmic vacuoles which contain the round, fragmented mitochondria (red area). The scale bar is 0.2 µm. (n = 3 images from 3 biological replicates). (B) isogenic ctrl-ECs displayed the reversal of the increased ROS in FD-ECs, as measured using DCFDA. Data represented as mean ±SD, n=3. Statistical significance of the observed changes was assessed with t-test *$p < 0.05$; ***$p < 0.005$; ****$p < 0.001$. ROS inducer is Pyocyanin, Pyo; ROS inhibitor is N-acetyl-L-cysteine, L-NAC.

Supplemental Table 1. Summary of Healthy Controls and Patients' cell lines Used in this study.

Table 1. Summary of Healthy Controls and Patients' cell lines Used in this study

Cells name	Age When Sample taken(Years)	*GLA* Genotype	Status	Sample Type	Isogenic control cell line
HUVECs	-	*Normal*	*Normal*	-	-
NTA	38	*Normal*	*Normal*	*Blood sample*	-
Isogenic ctrl1	-	*Normal*	*Normal*	*Non-viral transfection*	*FD1*
Isogenic ctrl2	-	*Normal*	*Normal*	*Non-viral transfection*	*FD2*
FD1	56	*IVS4+919G>A*	*Cardiac variant of Fabry disease*	*Skin biopsy*	-
FD2	48	*IVS4+919G>A*	*Cardiac variant of Fabry disease*	*Blood sample*	-

Supplemental Table 2. Sequences of the primers used for Probe, RT-PCR, and qPCR

**Name**	**Sequence**	**Predicted size**
sqRNA-GLA	F_TAGGCAGGTGGGATATCAGG<br>R_TTGCACTTGGAATGAAACCA	544
ddPCR	F_CACACTATTTGGAAGTATTTG<br>R_GAGAGATACAGTCAAAGTC	200
ddPCR Probe	5'-TGTCTCCCCACTAGAGTGTAAGTTTC-3'	
GLA 217	F_GTCCTTGGCCCTGAATAG<br>R_GTCCAGCAACATCAACAATT	217
GLA qPCR	F_TTGGATACTACGACATTGATGCC<br>q1R _GTATAATTGGGCTTTTGAAAGG<br>q2R_TAGTGGGGAGACATGGTAACAA	200
KLF4	F_ATGCTCACCCCACCTTCTTC<br>R_TTCTCACCTGTGTGGGTTCG	200
Ctgf	F_GGACCACATCTACGCTGACA<br>R_TTGACTGTGATCGGCTTCCC	184
GAPDH	F_AGAAGGCTGGGGCTCATTTG<br>R_AGGGGCCATCCACAGTCTTC	258
NANOG	F_CGTAAGCAGAAGAGGATCACC<br>R_GCTTCCTCCACCCACTTCTGC	179
PECAM1	F_AGGTCAGCAGCATCGTGGTCAACAT<br>R_GTGGGGTTGTCTTTGAATACCGCAG	187
KDR	F_TGCAAGGACCAAGGAGACTATGT<br>R_TAGGATGATGACAAGAAGTAGCC	458
vWF	F_GTTCGTCCTGGAAGGATCGG<br>R_CACTGACACCGTAGTGAGAC	168
CCL2	F_GATCTCAGTGCAGAGGCTCG<br>R_TGCTTGTCCAGGTGGTCCAT	152
CCL5	F_GCTGTCATCCTCATTGCTACTG<br>R_TGGTGTAGAAATACTCCTTGATGTG	129
CXCL1	F_GAAAGCTTGCCTCAATCCTG<br>R_CTTCCTCCTCCCTTCTGGTC	97
CXCL10	F_TGCCATTCTGATTTGCTGCC<br>R_TGCAGGTACAGCGTACAGTT	192
ICAM1	F_GGCCGGCCAGCTTATACAC	159

	R_TAGACACTTGAGCTCGGGCA	
IL6	F_AACCTGAACCTTCCAAAGATGG<br>R_TCTGGCTTGTTCCTCACTACT	159
IL8	F_TTTTGCCAAGGAGTGCTAAAGA<br>R_AACCCTCTGCACCCAGTTTTC	194
MIF	F_CGCAGAACCGCTCCTACAG<br>R_GGAGTTGTTCCAGCCCACAT	105

Supplementary Table 3. Antibodies used in this study.

**Target**	**Source**	**Catalog number**
GLA	GeneTex	GTX101178
Nanog	Cell Signaling Technology	#4903
Oct4	Cell Signaling Technology	#2750
Tra1-81	abcam	Ab16289
Tra1-60	abcam	Ab16288
Nestin	Cell Signaling Technology	#4760
SMA	Cell Signaling Technology	#19245
AFP	Cell Signaling Technology	#4448
Cd31_FITC	abcam	Ab33858
VE-cadherin	Santa cruz Biotechnology	Sc-6458
Cd31/PECAM1	Santa cruz Biotechnology	Sc-71872
Gb3/CD77	abcam	Ab19795
LC3	Novus Biologicals	NB100-200
P62	Novus Biologicals	NBP1-48320
VCAM1	Cell Signaling Technology	#13662
GAPDH	Cell Signaling Technology	#2118
CD54/ICAM1	Cell Signaling Technology	#4915
NF-kB p65	Cell Signaling Technology	#8242
Ikkα	Cell Signaling Technology	#2682
IKKβ	Cell Signaling Technology	#2678
p-Ikkα/ IKKβ	Cell Signaling Technology	#2697
ERK	Cell Signaling Technology	#4695
p-ERK	Cell Signaling Technology	#4370
AKT	Santa cruz Biotechnology	SC-5298

p-AKT	Santa cruz Biotechnology	SC-52940
p38	Cell Signaling Technology	#9212
p-p38	Cell Signaling Technology	#4511
